# The influence of elastomeric ligatures pigmentation on smile aesthetics during orthodontic treatment

**DOI:** 10.1590/2177-6709.26.2.e2119199.oar

**Published:** 2021-05-17

**Authors:** Amanda Galindo Florêncio MIRANDA, Ana Paula Terossi de GODOI, Carolina Carmo de MENEZES, Mário VEDOVELLO, Giovana Cherubini VENEZIAN

**Affiliations:** 1 Universidade Maurício de Nassau, Departamento de Odontologia (Caruaru/PE, Brazil).; 2 Centro Universitário da Fundação Hermínio Ometto, Departamento de Odontologia (Araras/SP, Brazil).

**Keywords:** Orthodontic brackets, Elastomeric ligatures, Dental aesthetic

## Abstract

**Aim:**

To assess the influence of elastomeric ligatures, subjected to a previous *in vitro* pigmentation process using different substances, on smile aesthetics during orthodontic treatment, from the perception of students and professionals.

**Methods:**

Eight elastomeric ligatures of five commercial brands (3M/Unitek, American Orthodontics, Morelli, Ortho Technology, and Orthometric) (n=8) were immersed in coffee, Coca-Cola, and red wine for one minute per day, for 28 days; and another group of ligatures was immersed in artificial saliva. All samples were photographed and subsequently analyzed using the Adobe Photoshop software, by the RGB method. Afterwards, the pigmented ligatures were inserted in a patient wearing orthodontic brackets, and zoomed photographs of the smile were taken and presented to 40 evaluators, who filled in a satisfaction scale sheet to express their opinion on the smile aesthetics of each photograph. The color data were subjected to analysis of variance (ANOVA) and Tukey tests.

**Results:**

The substance with the highest pigmentation potential was coffee (*p*< 0.05) followed by red wine (*p*< 0.05). Comparison among the brands used in this study showed that American Orthodontics and Orthometric had the lowest degree of pigmentation when immersed in coffee and red wine (*p*< 0.05), respectively. However, the brand that showed the highest level of satisfaction among the evaluators was Ortho Technology.

**Conclusions:**

The presence of pigmented elastomeric ligatures affected smile aesthetics, when compared with the control group.

## RESUMO

**Objetivo:**

Avaliar a influência das ligaduras elásticas submetidas a um processo prévio de pigmentação *in vitro*, com diferentes substâncias, na estética do sorriso durante o tratamento ortodôntico, segundo a percepção de alunos e profissionais.

**Métodos:**

Oito ligaduras elásticas de cinco marcas comerciais (3M Unitek, American Orthodontics, Morelli, Ortho Technology e Orthometric) (n = 8) foram imersas em café, Coca-Cola e vinho tinto por um minuto por dia, por 28 dias, e outro grupo de ligaduras foi imerso em saliva artificial. Todas as amostras foram fotografadas e posteriormente analisadas no *software* Adobe Photoshop, pelo método RGB. Em seguida, as ligaduras pigmentadas foram inseridas em um paciente com braquetes ortodônticos, e fotografias ampliadas do sorriso foram tiradas e apresentadas a 40 avaliadores, que preencheram uma escala de satisfação para representar sua opinião sobre a estética do sorriso de cada fotografia. Os dados de cor foram submetidos à análise de variância (ANOVA) e testes de Tukey.

**Resultados:**

A substância com maior potencial de pigmentação foi o café (*p*< 0,05), seguido do vinho tinto (*p*< 0,05). A comparação entre as marcas utilizadas nesse estudo mostrou que a American Orthodontics e a Orthometric apresentaram o menor grau de pigmentação quando imersas em café e vinho tinto (*p*< 0,05), respectivamente. Porém, a marca que apresentou maior nível de satisfação entre os avaliadores foi a Ortho Technology.

**Conclusões:**

A presença de ligaduras elásticas pigmentadas afetou a estética do sorriso, quando comparada à do grupo controle.

## INTRODUCTION

The increasing aesthetics concern has become an important issue into fixed orthodontic appliances, and has established it as a goal for orthodontists to achieve,^1^ especially during orthodontic treatment of adult patients.^2^ ^,^ ^3^ To meet the aesthetic expectations of more demanding patients during orthodontic treatment, aesthetic orthodontic appliances have been designed with the aim of filling this gap in the specialty.^4^ When offering these options to patients, orthodontists should use not only ceramic brackets, but accessories that also benefit this type of appliance, such as aesthetic elastic archwires and ligatures.^5^

The perception of the extent to which the ligature will harm smile aesthetics has a direct effect on the commercial brand selected by the orthodontist.^6^ Several studies^6-12^ have been conducted with the purpose of indicating to orthodontists the types of ligatures that have the lowest influence on aesthetics. However, the method used by the majority of studies analyzed was limited to laboratory tests,^6^ ^-^ ^10^ and overlooked the most important factor when assessing aesthetics; that is subjectivity, or evaluating only aesthetic perception without standardizing and assessing the degree of elastic pigmentation,^13^ making it impossible to identify the substances that most affect aesthetics. Therefore, further studies on pigmentation of elastomeric ligatures might be helpful in contributing to the improvement of these materials.^3^ In addition, this is relevant for professionals, as this will allow them to prioritize the use of materials with better characteristics and enable them to offer their patients guidance with regard to their diets. In this context, the present study aimed to assess the influence of aesthetic ligatures of five commercial brands on smile aesthetics during orthodontic treatment, from the perception of students and specialists. The ligatures were subjected to an *in vitro* pigmentation process with four solutions. This study tested the hypothesis that pigmentation of elastomeric ligatures would affect smile aesthetics and that there would be differences among the commercial brands and solutions tested.

## MATERIAL AND METHODS

The Ethics Committee of University Center of Hermínio Ometto Foundation (FHO) approved this study (number 2.125.443).

### SAMPLE SIZING

The sample calculation performed for the color stability analysis indicated that there was very low variability of samples among the elastomeric ligatures, thus providing a low coefficient of variation of 0.28% for the experiment. Therefore, the sample size of eight ligatures per group, resulting in 160 ligatures, provided a high degree of freedom in the residue analysis (DF=140), with test power higher than 0.99 for the main effects of brand and solution, and for the interaction between them.

For the aesthetic perception analysis, the sample of this study was calculated based on data from a previous study,^6^ which indicated coefficient of variation of 22.8% and sample size of 20 students and 20 orthodontists, resulting in 40 evaluators. This sample size resulted in a test power higher than 0.99 for the main effects of evaluator (student or orthodontist), brand, solution, and double or triple interactions.

### COLOR STABILITY ANALYSIS

Different commercial brands of aesthetic elastomeric ligatures were selected: 3M/Unitek (Monrovia, California, USA) - pearl, American Orthodontics (Sheboygan, Wisconsin, USA) - pearl, Morelli (Sorocaba, São Paulo, Brazil) - clear, Ortho Technology (Lutz, Florida, USA) - clear, and Orthometric (Marília, São Paulo, Brazil) - clear. Each group included eight ligatures (n=8) divided according to the five commercial brands and four solutions used for immersion, resulting in 160 ligatures. 

The *in vitro* pigmentation process was performed using the following four different potential solutions: coffee (Melitta, Minden, Louisiana, USA), Coca-Cola (Atlanta, Georgia, USA), red wine (Quinta do Morgado, Flores da Cunha, Rio Grande do Sul, Brazil) and artificial saliva (Saliform, Iasi, Romania).

**Figure 1:**
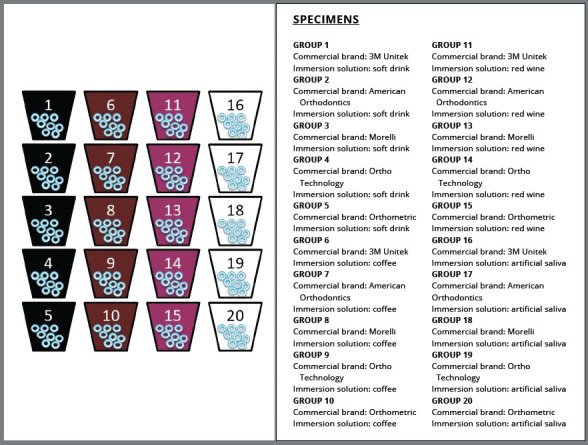
A sketch illustrating the specimens in each plastic receptacle identified by the number of the group, and the text on the right explaining both the commercial brand and the substance of immersion used in each group.

The ligatures were immersed for one minute per day in 40 ml of each solution to simulate the *in vivo* contact^14^ (Fig 1). After one minute, the specimens were inserted in containers containing artificial saliva, with one container for each group. These containers were wrapped in PVC plastic film and stored in an incubator at 37°C, to reproduce the intraoral temperature. The immersion solutions used were renewed every day. The specimens were photographed after 28 days, because the mean time interval between visits and consequent ligature replacement *in vivo* is known to be approximately 28 days.^1^ ^,^ ^6^ ^-^ ^13^ ^,^ ^15^ ^,^ ^16^

A digital camera (Canon EOS 70D model, Canon, Tokyo, Japan) with resolution of 20.2 megapixels, 16-bit compression, and 100-mm F-10 Canon lens was used to take the photographs. The camera was set at shutter speed of 1/50 seconds, diaphragm opening of 10, ISO 200, no flashlight, true color profile, and color temperature of 5000 K, with image captured in the RAW format and converted to the TIFF format.

This camera was attached to a support perpendicular to the object, at a distance of 31 centimeters above, in an environment free of natural and/or artificial light. The specimens were placed on a light table that was turned on and was the only light source in the room.^7^ A photograph was taken of all ligatures of each group after the in vitro pigmentation process, resulting in 160 photographs.

The images were transferred to the computer and converted to the TIFF format. Afterwards all the images were analyzed using the Adobe Photoshop CC 2017™ software (Adobe Systems Incorporated, San Jose, California, USA), in which a 100% zoom was applied for image visualization.^7^

**Figure 2:**
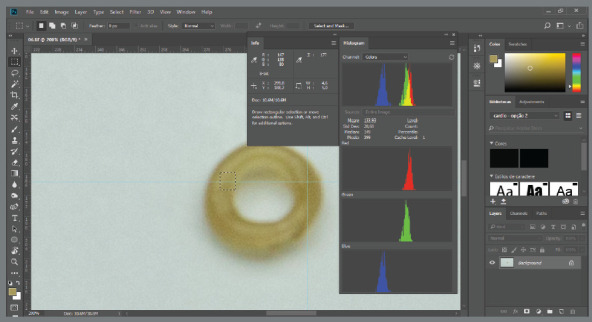
Image of color analysis of specimens in the Adobe Photoshop CC 2017^®^. The parameters of analyses by the RGB model are shown on the left side of the image.

The colors presented in the image were analyzed through levels of brightness (B), red (R), green (G), blue (B), and the sum of colors (RGB method). The color measured with the RGB method is aided by values in a scale ranging from 0 to 255, which provides the precise shade of a certain color. The closer the value was to 0, the darker would be the image. Similarly, the closer the value was to 255, the lighter would be the image (Fig 2), according to a previously published method.^7^ ^,^ ^9^

### AESTHETIC PERCEPTION ANALYSIS

**Figure 3:**
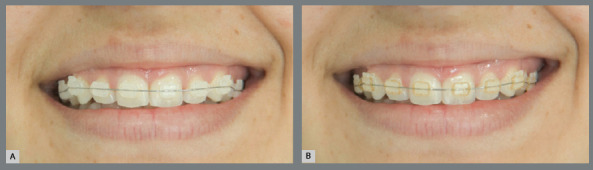
Photographs of the smile: (A) without pigmentation and (B) with pigmentation.

A patient wearing an aesthetic fixed orthodontic appliance with ceramic brackets from the Abzil brand, Transcend model, Roth prescription, 0.022-in slot, was selected as a model for the present study. The stages of dental alignment and leveling were concluded. All ligatures used in the first laboratory phase of this study were tied to the brackets already placed in the patient, using Mathieu tweezers. Photographs were taken of the patient’s smile in a constant position in all shots. To standardize the photographs, the patient was seated with Frankfurt and bipupilar planes parallel to the ground, the head was positioned with the help of head positioners and a cephalostat was placed on each side, to decrease movement as much as possible. The patient was instructed to keep smiling during the photographic shots. To prevent distortion of the images, the positioners were placed perpendicular to the camera lens, so that both right and left sides of the patient were at the same distance from the camera lens, following the method used by Ferraz et al.^6^ The same camera used in the laboratory phase was applied in the second phase, using a macro lens of 105 mm, with circular flashlight, and set at shutter speed of 1/50 seconds, with diaphragm opening of 8, true color profile, and color temperature of 5200 K. The camera was attached to a support perpendicular to the object, at a distance of 35 centimeters from the patient, using the ratio of 1:2.8 from the objective, to obtain a close-up photograph of the smile. Twenty photographs were taken, including the ligatures previously photographed in the laboratory phase. They were captured in the RAW format, transferred to and filed in a computer, in which they were converted to JPEG, printed at 2x magnification on 10x15 mm photographic paper, and identified with numbers from 1 to 20 (Fig 3).

A semi-structured questionnaire was prepared with 20 nominal satisfaction scales, with grades from 1 to 5, to determine the evaluator’s level of satisfaction with the smile aesthetics shown in each photograph, following the method proposed by Ferraz et al.^6^ Grade 1 corresponded to “very poor”, 2 was “poor”, 3 was “regular”, 4 was “good”, and 5 was “very good”, and the evaluators had to select the option most representative of their satisfaction.

For esthetic evaluation of the smile, all the photographs were judged by 40 evaluators (20 orthodontists and 20 dental students). The orthodontists aged between 27 and 51 years (mean of 37.15 years), with time since postgraduation ranging between 3 and 17 years (mean of 7.35 years). The professionals were randomly selected from a list of orthodontists registered at a Center of Post-Graduation in Dentistry (Random number calculators of the GraphPad Prism software program).

The other evaluators were students in the first year of dental school, aged between 18 and 35 years (mean of 24.55 years), randomly selected (Random number calculators of the GraphPad Prism software program). Students who had performed technical courses in the field of Dentistry were not included in the study. The selected students did not have the technical-scientific knowledge of an orthodontist and for the purpose of the present study they were considered lay persons.

### STATISTICAL ANALYSIS

After the descriptive and exploratory analysis, the color data (RGB model) were subjected to analysis of variance (ANOVA) in a 5x4 factorial scheme (brand x solution) and to the Tukey test. Data on the level of satisfaction were analyzed using generalized linear models by the PROC GENMOD procedure of the SAS software, considering the factors of brand, solution, and the interaction between them. All analyses considered a 5% level of significance.

### RESULTS

Table 1 shows that when immersed in artificial saliva (control group), the Orthometric ligatures showed the lightest color (226.00±0.53), followed by the brands Morelli (214.38±0.52), American Orthodontics (206.88±0.35), Ortho Technology (204.00±0.53), and lastly (the darkest ligatures) 3M/Unitek (192.63±0.74), with statistical difference among all of them (*p*<0.05).

**Table 1:** Mean color values (RGB method), mean (standard deviation), for both commercial brands and solution.

Commercial brand	Solution			
	Coke	Coffee	Red wine	Control group
3M Unitek	188.00 (0.53)^Bc^	136.88 (0.35)^Ce^	118.25 (0.46)^De^	192.63 (0.74)^Ae^
American Orthodontics	207.88 (0.35)^Aa^	202.88 (0.35)^Ba^	185.13 (0.35)^Cb^	206.88 (0.35)^Ac^
Morelli	178.38 (1.06)^Bd^	154.75 (0.46)^Dd^	158.00 (0.53)^Cd^	214.38 (0.52)^Ab^
Orto Technology	191.13 (0.35)^Bb^	163.00 (0.00)^Dc^	176.88 (0.35)^Cc^	204.00 (0.53)^Ad^
Orthometric	207.88 (0.64)^Ba^	194.13 (0.35)^Db^	196.63 (0.52)^Ca^	226.00 (0.53)^Aa^

Means followed by different letters (capital letters on the horizontal and lower case letters on the vertical) diverge among themselves (≤ 0.05).

Immersion in Coca-Cola showed a significant increase in pigmentation in all the groups, when compared with the control group (*p*≤0.05), except for American Orthodontics ligatures (207.88±0.35) (*p*>0.05). The lightest ligatures were those of American Orthodontics (207.88±0.35) and Orthometric (207.88±0.64) that showed no statistically significant difference between them (*p*>0.05). The darkest ligatures were those of Morelli (178.38±1.06) (*p*<0.05), which differed from all the other ligature brands.

When immersed in coffee, all brands also showed significantly lower mean pigmentation values than the control group (*p*<0.05), indicating that there was a significant increase in pigmentation. Furthermore, American Orthodontics ligatures (202.88±0.35) were the lightest (*p*<0.05), while the 3M/Unitek ligatures (136.88±0.35) were the darkest (*p*<0.05).

For the wine immersion solution, all brands also showed difference in color of one shade darker than the control group (*p*<0.05). Orthometric ligatures (196.63±0.52) were the lightest (*p*<0.05) and 3M/Unitek (118.25±0.46) ligatures were the darkest (*p*<0.05). 

**Figure 4:**
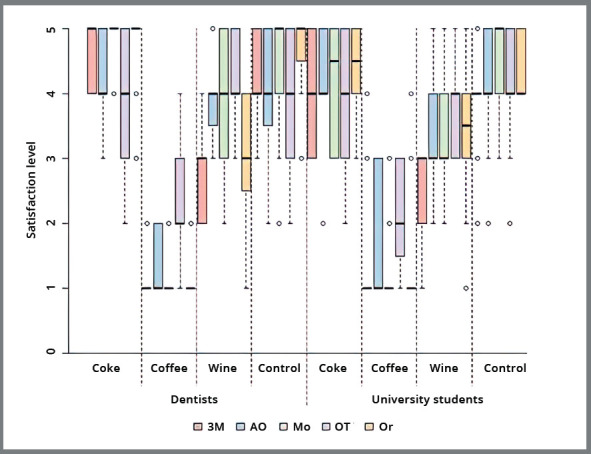
The box plot of the evaluator’s level of satisfaction with smile aesthetics relative to elastomeric ligatures of different commercial brands and the submersion solutions. Commercial brands: 3M/Unitek; AO (American Orthodontics); Mo (Morelli); OT (Ortho Technology); Or (Orthometric).

Figure 4 and Table 2 shows the results of the analysis on the level of satisfaction of orthodontists and university students with smile aesthetics. Both orthodontists and students showed a higher level of satisfaction with the photographs with ligatures immersed in artificial saliva (control group) and Coca-Cola, without differences between them (*p*>0.05).

**Table 2:** Median (minimum value; maximum value) of the evaluator’s level of satisfaction with the aesthetics of the smile according to the brand of elastomeric ligatures and the solution.

Evaluators	Brands	Solution			
		Coke	Coffee	Red wine	Control Group
Orthodontists	3M	*5.0 (4.0; 5.0)^Aab^	1.0 (1.0; 2.0)^Cb^	*3.0 (2.0; 4.0)^Bc^	4.0 (3.0; 5.0)^Aa^
	American Orthodontics	4.0 (3.0; 5.0)^Aab^	1.0 (1.0; 2.0)^Cb^	*4.0 (3.0; 5.0)^Bab^	4.0 (2.0; 5.0)^ABa^
	Morelli	*5.0 (4.0; 5.0)^Aab^	1.0 (1.0; 2.0)^Cb^	*4.0 (2.0; 5.0)^Bab^	5.0 (2.0; 5.0)^Aa^
	Orto Technology	4.0 (2.0; 5.0)^Ab^	2.0 (1.0; 4.0)^Ba^	4.0 (3.0; 5.0)^Aa^	4.0 (2.0; 5.0)^Aa^
	Orthometric	5.0 (3.0; 5.0) ^Aa^	1.0 (1.0; 2.0)^Cb^	3.0 (1.0; 4.0)^Bb^	5.0 (3.0; 5.0)^Aa^
University students	3M	4.0 (3.0; 5.0)^Aab^	1.0 (1.0; 4.0)^Cb^	3.0 (1.0; 3.0)^Bc^	4.0 (2.0; 5.0)^Aa^
	American Orthodontics	4.0 (2.0; 5.0)^Aab^	1.0 (1.0; 3.0)^Cb^	3.0 (2.0; 5.0) ^Bab^	4.0 (2.0; 5.0)^ABa^
	Morelli	4.5 (3.0; 5.0)^Aab^	1.0 (1.0; 3.0)^Cb^	3.0 (2.0; 5.0) ^Bab^	5.0 (3.0; 5.0)^Aa^
	Orto Technology	4.0 (2.0; 5.0)^Ab^	2.0 (1.0; 3.0)^Ba^	4.0 (3.0; 5.0)^Aa^	4.0 (2.0; 5.0)^Aa^
	Orthometric	4.5 (3.0; 5.0)^Aa^	1.0 (1.0; 4.0)^Cb^	3.5 (1.0; 5.0)^Bb^	4.0 (4.0; 5.0)^Aa^

*Significant differences among evaluators, under the same brand and solution conditions (p≤0.05). Medians followed by distinct letters (upper case comparing horizontally and lower case, vertically, compares brands within each solution and evaluator) differ (p≤0.05).

For Morelli, 3M/Unitek, and Ortho Technology, the level of satisfaction with the ligatures immersed in wine was lower, and even lower with those immersed in coffee (*p*<0.05). American Orthodontics and Ortho Technology showed significant difference in satisfaction of both orthodontists and students only with coffee (*p*<0.05).

For the ligatures immersed in Coca-Cola, a higher level of satisfaction by both orthodontists and students was perceived with the Orthometric (median=5) than with the Ortho Technology ligatures (median=4) (*p*<0.05). 

When immersed in coffee, all brands presented the worst level of satisfaction (median = 1) (*p*> 0.05) except for Ortho Technology ligatures (median = 2; poor) (p < 0.05). No difference between the evaluators (*p*> 0.05) was found. 

When immersed in wine, orthodontists showed higher satisfaction with the brands Ortho Technology, American Orthodontics, and Morelli (median = 4) than with Orthometric and 3M/Unitek (median = 3) (*p*< 0.05). For students, only Ortho Technology showed the highest level of satisfaction (median = 4).

In the majority of cases no significant difference was shown between orthodontists and students regarding level of satisfaction (*p*> 0.05). When a difference was found, the satisfaction of orthodontists reached higher values than the level of satisfaction of students (*p*< 0.05); this occurred for the brands 3M/Unitek and Morelli immersed in Coca-Cola and 3M/Unitek, American Orthodontics, and Morelli immersed in wine.

## DISCUSSION

The aesthetic importance of the smile has been extensively discussed in the literature.^2^ ^,^ ^17^ ^-^ ^19^ Orthodontics, a dental specialty that works directly with smile aesthetics, requires continuous updating of its material collection to keep up with the demands of the overall population.^4^ ^,^ ^5^ ^,^ ^20^ ^-^ ^24^

Therefore, testing the color change of elastomeric ligatures is required to guide orthodontists seeking to respect the decision of patients desiring an aesthetic smile even during orthodontic treatment. Therefore, several studies have been conducted^1^ ^,^ ^6^ ^-^ ^13^ ^,^ ^15^ ^,^ ^16^ with the aim of explaining which of the aesthetic elastomeric ligatures have the least effect on smile aesthetics.

In this context, *in vitro* studies that aim to simulate *in vivo* conditions controlled by laboratory researchers^14^ may be used to guide the clinical behavior. The laboratory studies conducted on the topic^1^ ^,^ ^6^ ^-^ ^12^ ^,^ ^15^ ^,^ ^16^ have immersed the specimens without establishing a time that would simulate the actual situation, ranging from 3 to 30 consecutive days of immersion. In order to simulate *in vivo* conditions more accurately, the present study determined that specimens would be immersed for only one minute per day, which is closer to the time individuals would be in contact with these solutions on a daily consumption basis.^14^ Moreover, in the literature consulted, few studies assessed such aesthetic perception.^1^ ^,^ ^6^ ^,^ ^13^ These studies did not include a laboratory stage,^13^ or did not measure color in the laboratory stage; the immersion solutions were mixed,^1^ ^,^ ^6^ and studies did not compare objective and subjective results, thus further studies with methodological variations are required for better explanation of the topic.

The literature also shows divergence regarding color analysis using a spectrophotometer^8^ ^,^ ^10^ ^,^ ^15^ ^,^ ^16^ and the Adobe Photoshop software.^7^ ^,^ ^9^ In the present study, analysis was performed with the Adobe Photoshop software due to its practicality and efficiency, as shown in previous studies.^7^ ^,^ ^9^

The laboratory results corroborated the findings of other studies,^6^ ^,^ ^7^ ^,^ ^9^ ^,^ ^10^ ^,^ ^12^ ^,^ ^13^ highlighting American Orthodontics ligatures as the most resistant to pigmentation when compared with the other brands analyzed, which suggested an association with the fabrication process. Other studies^1^ ^,^ ^11^ ^,^ ^16^ have indicated other brands such as GAC, Ormco, Orthodontic Supply, and TP Orthodontics as being the most aesthetic, but they did not use American Orthodontics ligatures, which prevents comparison with this brand.

As regards the brand of aesthetic elastomeric ligatures with the highest pigmentation values in the laboratory phase, the results found in the present study were similar to those of previous studies,^1^ ^,^ ^6^ ^,^ ^9^ ^,^ ^16^ indicating 3M/Unitek as the group of ligatures most susceptible to pigmentation when compared with the other brands analyzed. This may suggest that a higher level of pigmentation may be related to the fabrication process and even to a higher surface roughness of the ligature, so that further studies are required to analyze the fabrication process of the ligatures.

The subjective aesthetic perception using the satisfaction scale showed a consensus between the two groups of examiners, indicating Morelli as the least aesthetic brand and coffee as the solution with a higher pigmenting potential, which has been associated with the yellow pigment in its composition.^26^ However, Ortho Technology was the most aesthetic brand, in disagreement with all the laboratory results and confirming that aesthetics is in fact a subjective issue. Other studies have also found different results. Ferraz et al.^6^ indicated American Orthodontics and Morelli ligatures as being the most aesthetic and 3M/Unitek, Ortho Technology, and TP Orthodontics as the least aesthetic types. Talic and Almundhi^1^ corroborated the findings, also indicating 3M/Unitek, but they disagreed with the most aesthetic results, highlighting the Ormco brand. The study of Kawabata et al.^13^ confirmed Morelli as being the least aesthetic brand and 3M/Unitek, American Orthodontics, and GAC as the most aesthetic brands. This divergence of results may be associated with different methods, such as immersion time of the ligature in the pigmenting solution. In this study, orthodontists showed higher levels of satisfaction than students. Their clinical orthodontic experience and knowledge about the inevitable pigmentation of aesthetic ligatures are suggested to be factors that may have led to a more tolerant behavior relative to aesthetic perception by the orthodontists. 

The limitations highlighted in the present study are the non-differentiation between pearl and clear ligatures and the absence of pre-stretching before the pigmentation process, because evidence has shown that stretching the elastomers might affect the absorption of pigmenting agents.^9^ The authors suggest that further studies should be conducted, dissociating the types of ligatures and previous stretching in distinct groups. Also worth noting is that the elasticity degradation index was not tested in this study, requiring further studies comparing the data on color change with elasticity degradation, considering that the most aesthetic ligature is not necessarily the most effective one.

## CONCLUSIONS

Smile aesthetics was found to be affected by the presence of pigmented elastomeric ligatures. American Orthodontics and Orthometric elastomeric ligatures showed the lowest *in vitro* pigmentation levels when compared with the control group. The solution with the highest pigmenting potential was coffee.

Ortho Technology was found to be the brand of ligatures most favored by both groups of evaluators (orthodontists and students).
